# The Impact of Microbiome Interventions on the Progression and Severity of Inflammatory Bowel Disease: A Systematic Review

**DOI:** 10.7759/cureus.60786

**Published:** 2024-05-21

**Authors:** Malik Kasapoglu, Rajesh Yadavalli, Sarosh Nawaz, Abdulaziz Althwanay, Esraa M AlEdani, Harleen Kaur, Samia Butt

**Affiliations:** 1 Internal Medicine, California Institute of Behavioral Neurosciences & Psychology, Fairfield, USA; 2 Internal Medicine, Rajiv Gandhi Institute of Medical Sciences, Adilabad, IND; 3 Psychiatry, California Institute of Behavioral Neurosciences & Psychology, Fairfield, USA; 4 Dermatology, California Institute of Behavioral Neurosciences & Psychology, Fairfield, USA; 5 Medicine, Imam Abdulrahman Bin Faisal University, Dammam, SAU; 6 Medicine and Surgery, Maharishi Markandeshwar Institute of Medical Sciences and Research, Mullana, IND; 7 Research, California Institute of Behavioral Neurosciences & Psychology, Fairfield, USA

**Keywords:** crohn`s disease, fecal microbiota transplantation, inflammatory bowel disease, probiotics, synbiotics, ulcerative colitis

## Abstract

Inflammatory bowel disease (IBD), comprising Crohn's disease (CD) and ulcerative colitis (UC), is characterized by chronic intestinal inflammation. The dysbiotic gut microbiome likely contributes to IBD pathogenesis. Microbiome-directed therapies such as fecal microbiota transplantation (FMT), probiotics, and synbiotics may help induce and maintain remission. This systematic review aimed to determine the efficacy of microbiome interventions compared to standard therapy or placebo for IBD treatment. PubMed, EMBASE, Cochrane CENTRAL, and Web of Science were searched for randomized controlled trials on microbiome interventions in IBD from inception to October 2023. The risk of bias was assessed using Cochrane tools. Outcomes included disease activity, endoscopy, histology, quality of life, and adverse events. A total of 18 randomized controlled trials were included. Three trials found intensive (i.e., high frequency of administration and/or large volumes of fecal material) multi-donor FMT superior to autologous FMT or glucocorticoids for UC remission induction. Seven placebo-controlled trials demonstrated higher remission rates with FMT, especially intensive protocols, versus control for mild-to-moderate UC. However, a single FMT did not prevent relapses. Seven probiotic trials showed the potential to improve UC activity and maintain remission. One synbiotic trial reported reduced inflammation and symptoms versus placebo. Serious adverse events were rare. Small sample sizes and protocol heterogeneity limited the conclusions. Current evidence indicates the potential benefits of microbiome interventions, particularly intensive multi-donor FMT, for inducing and maintaining remission in UC. Probiotics may also improve outcomes. Adequately powered trials using standardized protocols are still needed to firmly establish efficacy and safety. Microbiome-directed therapies represent a promising approach for improving IBD outcomes.

## Introduction and background

Inflammatory bowel disease (IBD) is a term used to describe a group of chronic, idiopathic, inflammatory conditions of the gastrointestinal tract, primarily comprised of Crohn’s disease (CD) and ulcerative colitis (UC) [1,2]. IBD is characterized by alternating periods of remission and active intestinal inflammation, leading to symptoms such as abdominal pain, diarrhea, rectal bleeding, malnutrition, and fatigue [3-5]. The incidence and prevalence of IBD have steadily increased over the past 50 years, with over three million adults affected in the United States and Europe alone [6]. The substantial disease burden and lack of curative options highlight the need for safe and effective therapies to prolong remission [7].

The pathophysiology of IBD is believed to arise from a dysregulated immune response to the intestinal microbiota in genetically susceptible individuals [8,9]. This dysregulation involves an imbalance between proinflammatory and anti-inflammatory immune pathways, leading to chronic intestinal inflammation. In genetically predisposed individuals, environmental factors such as diet, infections, and antibiotic use can trigger or exacerbate this response. The gut microbiome plays a crucial role in immune system regulation, with dysbiosis (an imbalance in gut bacteria) exacerbating immune dysfunction in IBD [10]. Understanding this interplay provides the rationale for investigating microbiome-targeted therapies.

The management of IBD focuses on reducing inflammation and achieving remission. Treatments include medication, diet modifications, and sometimes surgery. Medications include aminosalicylates, corticosteroids, immunomodulators, and biological therapies such as anti-TNF agents [11,12]. Dietary changes, such as limiting dairy, caffeine, alcohol, and high-fiber foods, may help some [13]. In severe cases, surgery to remove the damaged portions of the intestine may be required [14]. Regular monitoring and adjustments to treatment are key to managing IBD long-term [15].

The human microbiome comprises over 100 trillion commensal bacteria, viruses, and fungi that interact with the gut mucosal immune system to maintain homeostasis [16]. The gut microbiome primarily consists of bacteria from the Firmicutes and Bacteroidetes phyla (both accounting for 90% of the gut microbiota), along with other groups such as Actinobacteria and Proteobacteria [17]. Patients with IBD typically exhibit reduced Firmicutes and increased Proteobacteria, leading to dysbiosis that may promote inflammation. Patients with IBD exhibit dysbiosis or an imbalance in the composition and function of their intestinal microbiome compared to healthy individuals [17]. Manipulating the gut microbiota through various microbiome interventions may modulate the enteric environment and immune responses in IBD [18].

There are several types of microbiome interventions, including fecal microbiota transplantation (FMT), probiotics, and synbiotics. FMT involves transferring healthy donor stool to a patient with IBD, aiming to restore a balanced and diverse microbiota [18]. This technique is intended to normalize the dysbiotic intestinal environment. Probiotics are live beneficial bacteria that can potentially suppress proinflammatory responses and enhance the gut's barrier function [19]. Different strains may have varying effects on IBD activity. Synbiotics combine probiotics with prebiotics, dietary fibers that promote the growth of beneficial bacteria. This combination may produce synergistic effects in reducing inflammation.

Emerging evidence suggests that microbiome-directed therapies may reduce intestinal inflammation, restore microbial diversity, and induce remission in IBD [18]. Probiotics, live beneficial bacteria, have shown a potential to dampen proinflammatory responses and improve epithelial barrier integrity [19]. Prebiotics, dietary fibers promoting the growth of commensal bacteria, may also confer anti-inflammatory effects [20]. FMT aims to normalize dysbiotic microbiota by transferring healthy donor stool to patients. While results appear promising, findings on the efficacy of microbiome interventions have been inconsistent across clinical trials [21-23].

A comprehensive synthesis of current evidence is needed to firmly establish the impact of microbiome-targeted therapies on the progression and severity of IBD. This systematic review aims to determine the effects of microbiome interventions compared to standard medical treatment or placebo on disease activity, endoscopic and histologic inflammation, quality of life, and adverse events in patients with CD, UC, and IBD unclassified.

## Review

Methods

The Cochrane Handbook for Systematic Reviews and Interventions [24] and Preferred Reporting Items for Systematic Reviews and Meta-Analyses (PRISMA) standards [25] were adhered to throughout this meta-analysis.

Literature Search and Search Strategy

A comprehensive literature search was performed to identify all relevant studies on microbiome interventions for IBD. The primary sources were PubMed/MEDLINE, EMBASE, Cochrane Central Register of Controlled Trials (CENTRAL), and Web of Science databases. The search was limited to human studies reported in English from database inception to October 2023. In our search strategy, we utilized the Medical Subject Headings (MeSH) terms provided below, combined with appropriate Boolean operators (OR, AND) and parentheses, to create focused search strings. By employing these MeSH terms and Boolean operators, we aimed to ensure a comprehensive and structured search approach: (1) ("Inflammatory Bowel Diseases"(Mesh)) OR ("Crohn Disease"(Mesh)) OR ("Colitis, Ulcerative"(Mesh)) OR ("Inflammatory Bowel Diseases, Unclassified"(Mesh)); (2) ("Microbiota"(Mesh)) OR ("Gastrointestinal Microbiome"(Mesh)) OR ("Gut Microbiota"(Mesh)) OR ("Intestinal Microbiota"(Mesh)); and (3) ("Probiotics"(Mesh)) OR ("Prebiotics"(Mesh)) OR ("Symbiotics"(Mesh)) OR ("Fecal Microbiota Transplantation"(Mesh)).

Eligibility Criteria

In this systematic review, the studies were selected based on predefined eligibility criteria. The participants included adults aged 18 years or older who had been diagnosed with CD, UC, or IBD unclassified according to standard criteria. Studies involving pediatric populations were excluded from the analysis.

The interventions considered in the review encompassed a wide range of microbiome-directed therapies, including probiotics, prebiotics, symbiotics, and FMT. The comparators in the selected studies included placebo, no treatment, standard IBD therapy, or active control groups. The outcomes of interest comprised various parameters, including disease activity scores, endoscopic and histologic scores, fecal calprotectin levels, C-reactive protein (CRP) levels, quality of life scores, and adverse events associated with the interventions.

The study design was limited to randomized and quasi-randomized controlled trials (RCTs). Studies with single-arm designs, case reports, conference abstracts, reviews, and ongoing trials were excluded from our review. No restrictions were imposed on the publication date, setting, or duration of the interventions, allowing for a comprehensive evaluation of the available literature.

Selection Criteria

Records were transferred into EndNote X9 and exported to an MS Excel spreadsheet (Microsoft Corporation, Redmond, Washington). First, two reviewers independently screened the titles and abstracts of the retrieved studies for potential eligibility. Next, full texts of potentially relevant studies were assessed for final inclusion. Any discrepancies will be resolved by discussion and consensus with a third reviewer. The reference lists of included studies and relevant systematic reviews were examined for additional potential studies.

Data Extraction

One reviewer extracted the data from the included studies into a standardized form in Covidence, while a second reviewer will verify the extracted data for accuracy and completeness. Information to be extracted consists of the following: (1) study characteristics (author, year, country, design) and (2) participant characteristics (sample size, age, sex, Mayo score). The Mayo score is an index of disease activity commonly used in placebo-controlled trials in UC. It contains four domains: rectal bleeding, stool frequency, physician assessment, and endoscopy appearance. Each domain is given a score of 0 to 3, with an overall score ranging from 0 to 12.

Risk of Bias Assessment

In this systematic review, the methodological quality of the selected studies was rigorously evaluated using the Cochrane Risk of Bias tool - Version 2 [26]. Two independent reviewers conducted a comprehensive assessment of RCTs, focusing on six key domains: bias arising from the randomization process, deviations from intended interventions, missing outcome data, measurement of outcomes, selection of the reported result, and overall risk of bias. Each study was categorized as having either a low risk of bias, some concerns, or a high risk of bias based on the assessments within these domains. By employing this robust methodology, we ensured a thorough evaluation of the included studies, allowing for a nuanced understanding of the impact of microbiome interventions on the progression and severity of IBD.

Data Synthesis

The extracted data were analyzed qualitatively using a narrative approach. The conduct of quantitative synthesis (i.e., meta-analysis) was not feasible given the heterogeneous nature of the included populations, examined interventions, and outcome reporting methods.

Results

**Figure 1:**
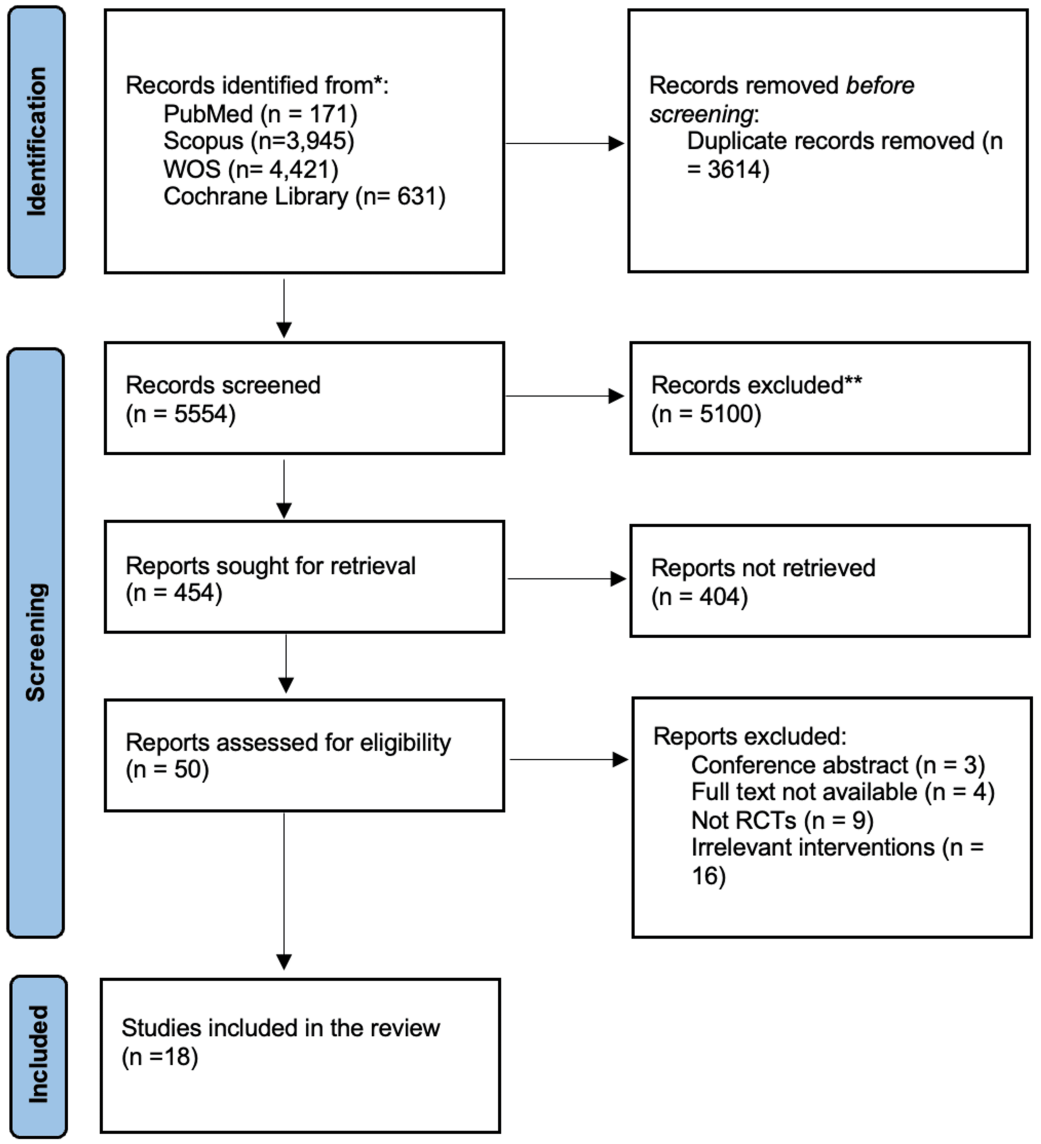
A PRISMA flow diagram showing the results of the systematic literature search *Number of citations retrieved from each database; **number of citations excluded following the title and abstract screening phases n: number of citations; WOS: Web of Science; RCTs: randomized controlled trials; PRISMA: Preferred Reporting Items for Systematic Reviews and Meta-Analyses

A comprehensive literature search of major databases (PubMed, Scopus, Web of Science, Cochrane Library) yielded 9168 records. After removing 3614 duplicate reports, 5554 records remained for screening. The title and abstract screening excluded 5100 reports as irrelevant, leaving 454 reports for full-text retrieval. However, full texts could not be obtained for 404 reports. The remaining 50 reports underwent full-text review, of which 18 RCTs fulfilled the eligibility criteria and were included in the systematic review (Figure 1) [27-44].

Baseline and Characteristics of the Included Studies

The sample sizes in each group ranged from 6 to 173 participants. The mean or median ages were in the 30s-40s range for most studies. The majority of studies had slightly more male than female participants. Baseline disease severity assessed by Mayo scores was in the moderate to severe range (scores 5-9) for UC patients. The mean disease duration was 5-10 years in most UC trials (Table 1).

**Table 1:** Summary of the included studies RCT: randomized controlled trial; UC: ulcerative colitis; CD: Crohn’s disease; FMT: fecal microbiota transplantation; UCDAI: Ulcerative Colitis Disease Activity Index; DC: dendritic cell; NR: not reported; USA: United States of America; UK: United Kingdom; IBD: inflammatory bowel disease; Formulation De Simone: a proprietary mixture of *Lactobacillus acidophilus*, *Lactobacillus bulgaricus*, *Lactobacillus casei*, *Lactobacillus plantarum*, *Bifidobacterium breve*, *Bifidobacterium infantis*, *Bifidobacterium longum*, and *Streptococcus salivarius* ssp *thermophilus*

Study ID	Site	Study Design	Study Period	Patients	Intervention	Control	Follow-up	Primary Outcomes	Conclusion
FMT vs. active control									
Schierová et al. 2020 [42]	Czech Republic	RCT	NR	Active left-sided UC	Donor FMT	Aminosalicylates	6 weeks	Clinical remission	FMT was promising for the UC
Rossen et al. 2015 [43]	Netherlands	RCT	2011-2014	Active, mild to moderate UC	Donor FMT	Autologous FMT	12 weeks	Clinical and endoscopic remission	There was no difference in remission rates
Costello et al. 2019 [44]	Australia	RCT	2013-2016	Active UC	Anaerobically prepared pooled donor FMT	Autologous FMT	12 months	Steroid-free remission	Donor FMT increased remission likelihood
FMT vs. control									
Lahtinen et al. 2023 [35]	Finland	RCT	2016-2020	Active UC	Donor FMT	Control	12 months	Maintenance of remission	There were no differences in relapses
Crothers et al. 2021 [36]	USA	RCT	2015-2018	Active UC	FMT plus encapsulated oral FMT	Sham therapy	12 weeks	Safety outcomes	Encapsulated FMT had extended effects
Fang et al. 2021 [37]	China	RCT	2017-2021	Recurrent active UC	Donor FMT	Control	8 weeks	Steroid-free remission	FMT restored microbiota composition
Sokol et al. 2020 [38]	France	RCT	2014-2017	Active CD	Donor FMT	Control	6 weeks	Steroid-free remission	No primary endpoint achieved
Moayyedi et al. 2015 [39]	Canada	RCT	2012-2014	Active UC	Donor FMT	Control	6 weeks	UC remission	FMT increased remission
Paramsothy et al. 2017 [40]	Australia	RCT	2013-2015	Active UC	Donor FMT	Control	8 weeks	UC remission	Multiple FMT-induced remission
Sood et al. 2019 [41]	India	RCT	2016-2018	Active UC	Donor FMT	Control	48 weeks	Relapse-free survival	FMT maintained remission
Probiotic									
Ng et al. 2010 [27]	UK	RCT	NR	Mild to moderate UC	Formulation De Simone	Control	8 weeks	Acute phase reactants	Formulation De Simone improved the DC function
Tursi et al. 2010 [28]	Italy	RCT	NR	Mild to moderate UC	Formulation De Simone	Control	8 weeks	UCDAI scores	Formulation De Simone decreased UCDAI scores
Matsuoka et al. 2018 [30]	Japan	RCT	2012-2013	Active UC	*Bifidobacterium breve *fermented milk	Control	48 weeks	Relapse-free survival	No impact on relapse time
Yasueda et al. 2015 [31]	Japan	RCT	2007-2013	Active UC	*Clostridium butyricum* (CBM)	Control	24 months	Pouchitis-free survival	CBM showed positive outcomes
Yoshimatsu et al. 2015 [32]	Japan	RCT	NR	Inactive UC	Bio-Three tablets, each containing lactomin, *Clostridium butyricum*, and *Bacillus mesentericus*	Control	12 months	Relapse rate	Probiotics showed sustained remission
Tamaki et al. 2015 [33]	Japan	RCT	NR	Mild to moderate UC	*Bifidobacterium longum* 536	Control	8 weeks	Clinical remission	BB536 reduced activity
Shadnoush et al. 2015 [34]	Iran	RCT	NR	Active IBD	Probiotic yogurt	Control	8 weeks	Weight and body mass index	Probiotics improved function
Synbiotic									
Kamarlı et al. 2019 [29]	Turkey	RCT	2016-2017	Mild to moderate UC	Six probiotic strains and fructooligosaccharide, which is a prebiotic fiber	Control	8 weeks	Acute phase reactants and clinical and endoscopic activities	Synbiotic improved activity

The studies compared FMT to autologous FMT or placebo, probiotics to placebo, and one synbiotic to placebo. Follow-up durations ranged from six weeks to two years. Primary outcomes were various measures of disease remission, relapse, or activity scores (Table 2).

**Table 2:** Baseline characteristics of the included studies #median and ranges; *median and interquartile ranges FMT: fecal microbiota transplantation

Study ID	Group	Sample	Age, Years, Mean (SD)	Sex, Male (%)	Total Mayo Score, M (SD)	Duration of Disease, M (SD)
FMT vs. active control						
Schierová et al. 2020 [42]	Donor FMT	8	37.5 (28–62)*	4 (50)	5.5 (4–9)*	-
	Aminosalicylates	8	40 (31–66)*	4 (50)	5.5 (3–9)*	-
Rossen et al. 2015 [43]	Donor FMT	23	-	-	-	-
	Autologous FMT	25	-	-	-	-
Costello et al. 2019 [44]	Donor FMT	38	38.5 (28-52)#	20 (53)	7.2 (1.7)	4.9 (1.6-9.6)#
	Autologous FMT	35	35 (25-46)#	20 (57)	7.4 (1.9)	5.8 (2.4-11)#
FMT Vs. control						
Lahtinen et al. 2023 [35]	Donor FMT	24	43.0 (12.9)	14 (58.3)	-	3.3 (4.25)
	Control	24	43.1 (12.1)	12 (50)	-	9.5 (9.8)
Crothers et al. 2021 [36]	FMT plus encapsulated oral FMT	6	41 (15)	4 (67)	6.3 (2.0)	8.9 (9.1)
	Sham therapy	6	52 (15)	3 (50)	6.7 (1.2)	9.8 (10.6)
Fang et al. 2021 [37]	Donor FMT	10	51.5 (12.7)	8 (80)	9.5 (2.5)	5.9 (7.3)
	Control	10	44.6 (14.9)	8 (80)	8.6 (2.9)	6.9 (9.3)
Sokol et al. 2020 [38]	Donor FMT	8	31.5 (27.5-36.5)#	5 (62.5)	4 (8.5-12.8)#	-
	Control	9	34 (33-52)#	4 (44.4)	8 (11-15)#	-
Moayyedi et al. 2015 [39]	Donor FMT	38	42.2 (15)	18 (47)	8.24 (2.61)	7.9 (5.6)
	Control	37	35.8 (12.1)	26 (70)	7.86 (2.28)	7 (6.8)
Paramsothy et al. 2017 [40]	Donor FMT	41	35.6 (27.8–48.9)#	22 (54)	5.8 (3.4–9.0)#	8 (6–9)#
	Control	40	35.4 (27.7–45.6)#	25 (63)	5.8 (2.7–9.4)#	8 (6–9)#
Sood et al. 2019 [41]	Donor FMT	31	33 (12.4)	22 (70.9)	1.9 (0.3)	4.1 (2.9)
	Control	30	34.6 (12.3)	22 (73.3)	1.8 (0.4)	4.5 (3.5)
Probiotic vs. control						
Ng et al. 2010 [27]	Formulation De Simone	14	45 (21–70) *	4 (28.6)	-	5 (1–16)*
	Control	14	41 (28–57)*	7 (50)	-	7 (1–27)*
Tursi et al. 2010 [28]	Formulation De Simone	71	47.7 (14.1)	49 (69)	-	-
	Control	73	46.4 (14.4)	44 (60.3)	-	-
Matsuoka et al. 2018 [30]	*Bifidobacterium breve* strain	97	41.3 (20–70)*	50 (51.5)	-	-
	Control	95	41.8 (20–66)*	50 (52.6)	-	-
Yasueda et al. 2015 [31]	Clostridium butyricum	9	47 (25)	5 (55.6)	-	5 (24)
	Control	8	34 (48)	4 (50)	-	1.5 (15.5)
Yoshimatsu et al. 2015 [32]	Bio-Three tablets	23	44.8 (13.8)	16 (70)	-	8 (6.3)
	Control	23	42.9 (15.9)	12 (52)	-	6.7 (5.9)
Tamaki et al. 2015 [33]	*Bifidobacterium longum* 536	28	44.9 (14.5)	-	-	-
	Control	28	45.5 (13.8)	-	-	-
Shadnoush et al. 2015 [34]	Probiotic yogurt	86	36.6 (9.1)	54 (62.8)	-	10.6 (5.8)
	Control	90	37.7 (8)	50 (55.6)	-	11.6 (5.1)
Synbiotic						
Kamarlı et al. 2019 [29]	Synbiotic	18	44.9 (14.1)	9 (50)	-	4.67 (6.23)
	Control	18	40 (12.7)	10 (55.6)	-	4.6 (4.4)

Quality Assessment

**Figure 2:**
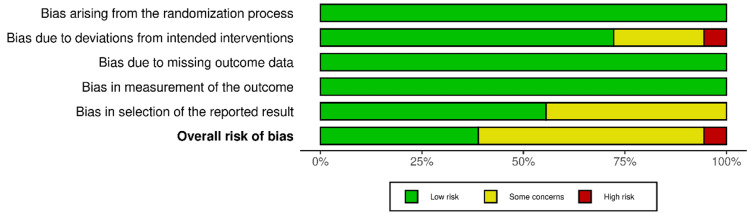
Graph showing the summary of the overall risk of bias of the included RCTs using Cochrane's RoB-2 tool RCTs: randomized controlled trials; RoB-2: risk of bias - version 2

**Figure 3:**
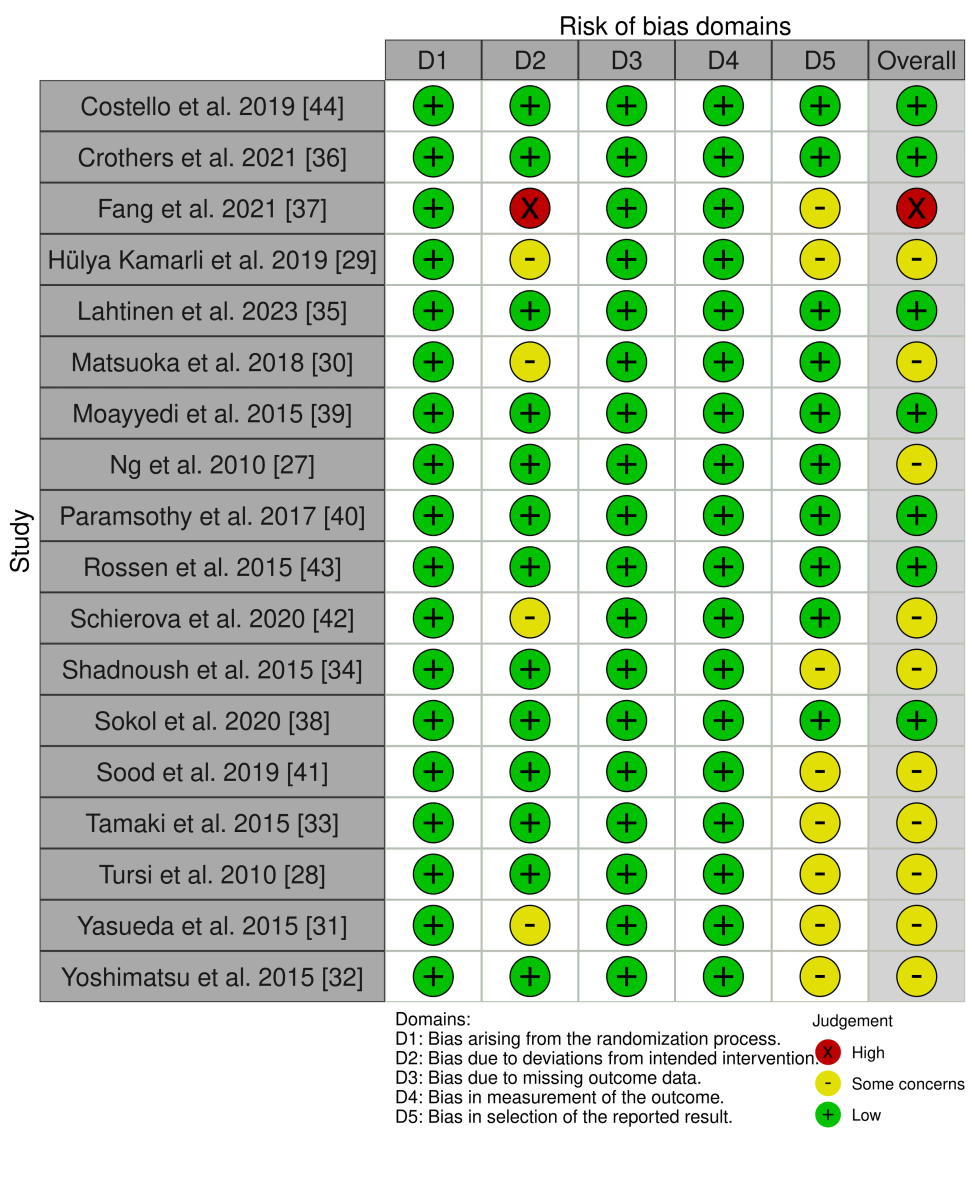
Graph showing the risk of bias of the examined RCTs across the different domains of the Cochrane's RoB-2 tool RCTs: randomized controlled trials; RoB-2: risk of bias - version 2

The overall risk of bias for each study was determined based on the assessments across the five domains. Seven studies were rated as having a low risk of bias overall [35,36,38-40,43,44], with 10 studies showing concerns in one or more domains, leading to an overall rating of some concerns [27-34,41,42]. Only one study, Fang et al. 2021, was rated a high risk of bias overall due to a high risk of bias in one domain (Figures 2, 3) [37].

FMT Versus Active Controls

Three RCTs have compared FMT to an active control for remission induction in IBD. Costello et al. (2019) conducted a multicenter trial comparing anaerobically prepared pooled donor FMT (n=38) to autologous FMT (n=35) via colonoscopy and enemas over seven days [44]. Although the stool containers of both groups had identical ratios and volumes of stool, saline, and glycerol, the pooled donor stool was prepared anaerobically, while the autologous stool was processed under aerobic conditions. During the colonoscopy, 200 mL of fecal suspension of either donor stool or autologous stool was delivered into the right colon. The FMT group had significantly higher rates of steroid-free remission at eight weeks than autologous FMT (32% vs. 9%, p=0.03). Adverse events were similar between groups.

Schierová et al. (2020) conducted a small trial comparing FMT to 5-aminosalicylic acid (5-ASA) enemas for left-sided UC [42]. Remission occurred in 37.5% of FMT patients and 50% of 5-ASA patients. In the FMT group, remission was associated with increased abundance of beneficial Firmicutes and Actinobacteria families and genera, including Faecalibacterium, Blautia, and Bifidobacterium, suggesting that FMT can alter microbiota composition in a potentially therapeutic way.

Finally, Rossen et al. (2015) compared two doses of FMT from healthy donors (n=23) to autologous FMT (n=25) via nasoduodenal tube [43]. No significant difference was seen in the composite endpoint of clinical and endoscopic remission at 12 weeks between FMT and control groups (30% vs. 20%, p=0.51). FMT responder microbiota became more similar to donors, and remission was associated with increased Clostridium clusters.

FMT Versus Placebo Controls

Seven randomized placebo-controlled trials have evaluated FMT for UC treatment. Crothers et al. (2021) performed a pilot trial of single colonoscopic FMT followed by 12 weeks of daily encapsulated FMT (n=6) versus sham therapy (n=6) [36]. In the sham group, the subjects received sham colonoscopic infusion with sham capsules, which were designed to visually resemble fecal material. Two FMT patients achieved remission versus none in the control group. FMT induced sustained microbiome changes.

Several trials have assessed single FMT for induction of remission. Fang et al. (2021) reported higher remission rates at eight weeks for single FMT (90%, n=10) compared to control (50%, n=10) in mild-to-moderate UC [37]. However, Lahtinen et al. (2023) found no difference in one-year relapse rates between single FMT (n=24) and autologous transplant (n=24) [35].

Other trials have evaluated intensive, multi-dose FMT protocols. Moayyedi et al. (2015) found six weekly FMT enemas (n=38) superior to placebo (n=37) for inducing remission at seven weeks (24% vs. 5%) [39]. Paramsothy et al. (2017) also reported higher rates of remission at eight weeks with intensive FMT enemas (n=41) versus placebo enemas (n=40) (27% vs. 8%) [40].

For maintenance of remission, Sood et al. (2019) reported no difference in one-year clinical remission between maintenance FMT (n=31) and placebo (n=30) when added to standard UC therapy [41]. However, secondary endpoints of endoscopic and histologic remission were higher with FMT.

However, in terms of CD, only one trial compared the effect of FMT to placebo control. The study by Sokol et al. (2019) compared FMT (eight patients) to sham transplantation (nine patients) in adults with CD [38]. The primary endpoint was the implantation of donor microbiota (Sorensen index > 0.6), assessed at six weeks. Although none of the patients reached this primary endpoint, at 10 weeks, the FMT group exhibited a higher steroid-free clinical remission rate (87.5%) compared to the sham group (44.4%). The authors used the Crohn's Disease Activity Index (CDAI) to determine clinical flare (with scores > 220), which tended to be higher in the sham group (66.66%) compared to the FMT group (37.5%).

Probiotics

Seven RCTs have evaluated probiotics for UC treatment. Matsuoka et al. (2018) found no difference in 48-week relapse rates between *Bifidobacterium breve* and *Lactobacillus acidophilus* fermented milk (n=98) versus placebo (n=97) as maintenance therapy [30].

Four trials assessed probiotics for inducing remission in mild-to-moderate UC. Ng et al. (2010) reported immunomodulatory effects and a 71% clinical response rate with Formulation De Simone (n=14) compared to 36% for placebo (n=14) [27]. Tamaki et al. (2015) found improved disease activity with *Bifidobacterium breve* (n=28) versus placebo (n=28) [33]. Shadnoush et al. (2015) reported increased fecal Lactobacillus, Bifidobacterium, and Bacteroides after probiotic yogurt (n=105) compared to placebo (n=105) [34]. Lastly, Tursi et al. (2010) found Formulation De Simone (n=71) superior to placebo (n=73) for reducing UC disease activity scores [28].

For maintenance of remission, Yoshimatsu et al. (2015) reported lower relapse rates at three and six months with Bio-Three probiotics (n=23) versus placebo (n=23) [32]. Yasueda et al. (2015) also found fewer cases of pouchitis with probiotics (n=9) compared to placebo (n=8) [31].

Synbiotics

Only one randomized placebo-controlled trial by Kamarlı (2019) evaluated a synbiotic for UC [29]. Forty patients received either synbiotics (n=20) or placebo (n=20) for eight weeks along with standard therapy. The synbiotic group had significant decreases in CRP and sedimentation rate (p=0.003), along with significantly greater improvement in clinical disease activity compared to placebo (p<0.05). However, no significant difference was seen in endoscopic disease activity.

Discussion

This systematic review synthesized evidence from 18 RCTs evaluating the efficacy of microbiome interventions, including FMT, probiotics, and synbiotics, for IBD treatment. Overall, the results suggest the potential benefits of these therapies for inducing and maintaining disease remission, although small sample sizes and some methodological issues limit definitive conclusions.

FMT aims to restore diversity in the dysbiotic IBD microbiome. Our review found FMT consistently more effective than autologous FMT or glucocorticoids for remission induction in UC, highlighting its potential as an alternative to standard medications. Intensive, multi-donor FMT protocols appeared most efficacious. The superiority of pooled donor FMT implies that transferring a highly diverse fecal microbiota is important for reconstituting microbial communities in IBD [42-44].

Mechanistically, an increased abundance of potentially beneficial bacterial taxa such as Faecalibacterium, Bifidobacterium, and Clostridium clusters IV and XIVa were associated with FMT response. These bacteria likely exert positive immunomodulatory effects through the production of short-chain fatty acids and other anti-inflammatory metabolites. However, a single FMT showed no benefit in the maintenance of remission, indicating a need for ongoing microbial exposure to sustain changes. Encouragingly, no major safety issues arose with FMT.

While initial results are promising, adequately powered sham-controlled trials using standardized protocols are still required to definitively determine the efficacy of FMT for the induction and maintenance treatment of IBD. Microbiome and immunological analyses in future studies may help identify predictors of FMT response [35-37,39].

Several RCTs in our review evaluated probiotics, live commensal bacteria, for UC management. Overall, probiotics demonstrated the potential to improve disease activity and maintain remission, especially in milder UC. Benefits may occur through enhancing mucosal barrier function and downregulating inflammatory cytokine production. However, due to modest sample sizes, differences versus placebo did not reach statistical significance in most studies. In mouse models, the bacterium *Faecalibacterium prausnitzii*, belonging to the Firmicutes phylum, exhibits notable anti-inflammatory effects by secreting metabolites that diminish inflammatory cytokine (i.e., interleukin (IL)-1 β, IL-12, and interferon-gamma (IFN-γ) production, thereby preventing active colitis [45]. Commensal organisms also promote anti-inflammatory responses through mechanisms such as activating regulatory CD4 T-cells and stimulating anti-inflammatory cytokine production [46,47]. However, in the case of UC, both the response and non-response groups to FMT did not exhibit a difference in any of the examined 11 cytokines (IL-1β, IL-2, IL-4, IL-6, IL-10, IL-11, IL-17A, IFN-γ, tumor necrosis factor (TNF), TNF receptor (TNFR)-1, TNFR-2, monocyte chemoattractant protein (MCP)-1, granulocyte colony-stimulating factor (G-CSF), and granulocyte-macrophage colony-stimulating factor (GM-CSF) [48]. Optimal probiotic strains, dosages, and delivery methods remain unclear. No major safety issues arose with short-term use, but long-term adverse effects cannot be excluded, given limited follow-up durations [28,30-34].

Only one small trial examined a synbiotic, combining probiotics and prebiotics, for UC treatment. While results were promising, with significantly greater reductions in systemic inflammation and disease activity than placebo, confirmation in larger synbiotic trials is needed [29].

Several previous systematic reviews have also examined the efficacy of microbiome interventions for IBD treatment. A Cochrane review conducted by Iheozor-Ejiofor et al. (2020) reported that the impact of probiotics in sustaining remission for UC is uncertain due to the limited and unreliable evidence from poorly conducted studies. These studies provide minimal data from a small group of participants, making it challenging to draw definitive conclusions about the effectiveness of probiotics in this context [49]. In their 2021 study, Chen et al. conducted a systematic analysis of the effectiveness of probiotics in CD and UC. They discovered that probiotics can help induce remission in UC during its active phase. However, the study did not find significant therapeutic benefits in remission for CD and UC [50].

This systematic review has several important clinical implications. It suggests that microbiome-modulating therapies such as FMT and probiotics should be considered alongside standard IBD medications, especially for patients with mild-to-moderate UC [51,52]. Intensive, pooled donor FMT protocols appear most appropriate for inducing remission, while probiotics may help maintain remission [53]. Patients could be screened for potential biomarkers, such as fecal calprotectin or microbiome profiles, to determine suitability [54]. The reported good safety profiles support the inclusion of these interventions into IBD treatment guidelines. However, longer-term adverse effects require further study. For instance, the recent meta-analytic study of Li et al. (2015) showed that all of FMT’s adverse events were short-lived, moderate, and manageable, with death being reported in 38 out of 736 cases (5.16%) [55]. However, the authors used a cutoff point of 90 days to define long-term effects. This needs to be readjusted in future studies by extending the follow-up period beyond six months. Optimization of therapeutic protocols through adequately powered RCTs should be prioritized. As our understanding of IBD pathogenesis evolves, harnessing the microbiome's potential through rational manipulation offers an exciting avenue for improving long-term outcomes.

Strengths and Limitations

A key strength of this review was the comprehensive literature search across major databases using a robust strategy. Limiting inclusion to RCTs also allowed for a high-quality assessment of microbiome interventions for IBD. Strict adherence to PRISMA guidelines and Cochrane methodologies reduced risks of bias and errors in the review process. Nonetheless, conclusions must be interpreted cautiously in light of some limitations. One significant challenge was the exclusion of approximately 450 studies due to a lack of full-text availability. Although we took the initiative to reach out to the first and corresponding authors of these studies, we limited this outreach to articles published within the last 10 years to ensure that contact details were current. Authors were contacted via email or ResearchGate messages, but of the 391 authors contacted, 367 did not respond, and 21 refused to provide the full text. Although three authors initially agreed to share their data after obtaining institutional approval, follow-up attempts went unanswered. Additionally, major issues were small sample sizes in most trials and heterogeneity in probiotic strains, FMT protocols, and outcome measures, precluding meta-analysis. Many studies had short follow-up durations, providing little insight into long-term efficacy and safety. Finally, the majority of trials focused on UC, so extrapolation of findings to CD is difficult.

Future Research Directions

While current evidence indicates the potential benefits of microbiome-directed therapies for treating IBD, there are several gaps in the literature that future research should address to clarify their clinical utility. First, future studies should focus on developing standardized protocols for FMT and probiotics. This will allow comparisons across trials and provide more definitive data on optimal donor selection, FMT administration frequency, probiotic strains, dosages, and delivery methods. Creating a consistent framework for microbiome interventions will enhance the quality and reliability of the evidence. Second, many of the existing trials are limited by small sample sizes, which affects the statistical power of their findings. Larger, multicenter RCTs with robust designs are needed to establish the efficacy and safety of microbiome interventions for both induction and maintenance of remission. These trials should also include adequate control groups and employ sham or placebo procedures to minimize bias. Third, current studies often have short follow-up periods, limiting insights into the long-term efficacy and safety of these interventions. Longer-term studies are necessary to evaluate sustained outcomes, potential adverse effects, and the durability of benefits from microbiome therapies over time. Fourth, most existing research focuses on UC, with less attention given to CD. Future studies should include a balanced examination of both conditions to understand the broader applicability of microbiome interventions across the IBD spectrum. Finally, identifying reliable biomarkers, such as fecal calprotectin levels or specific microbiome profiles, can help predict patient responses to FMT and probiotics. Personalized approaches that screen patients for these biomarkers may improve patient selection and optimize outcomes.

## Conclusions

In summary, current evidence indicates the potential efficacy of microbiome-modulating therapies such as FMT, probiotics, and possibly synbiotics for inducing and maintaining IBD remission. Intensive, multi-donor FMT protocols appear most promising for UC management, based on the strongest RCT evidence. However, adequately powered, high-quality trials with extended follow-up are still required to establish the role of these interventions in IBD treatment. Optimization and standardization of therapeutic protocols will be essential. Careful mechanistic analyses may uncover microbiome, immunologic, and genetic factors that predict responses. As our understanding of the microbiome's contribution to IBD pathogenesis deepens, harnessing its restorative potential through microbiome-directed therapies offers an exciting avenue for improving long-term outcomes in IBD.
