# Correction: The Impact of Microbiome Interventions on the Progression and Severity of Inflammatory Bowel Disease: A Systematic Review

**DOI:** 10.7759/cureus.c205

**Published:** 2024-12-23

**Authors:** Malik Kasapoglu, Rajesh Yadavalli, Sarosh Nawaz, Abdulaziz Althwanay, Esraa M AlEdani, Harleen Kaur, Samia Butt

**Affiliations:** 1 Internal Medicine, California Institute of Behavioral Neurosciences & Psychology, Fairfield, USA; 2 Internal Medicine, Rajiv Gandhi Institute of Medical Sciences, Adilabad, IND; 3 Psychiatry, California Institute of Behavioral Neurosciences & Psychology, Fairfield, USA; 4 Dermatology, California Institute of Behavioral Neurosciences & Psychology, Fairfield, USA; 5 Medicine, Imam Abdulrahman Bin Faisal University, Dammam, SAU; 6 Medicine and Surgery, Maharishi Markandeshwar Institute of Medical Sciences and Research, Mullana, IND; 7 Research, California Institute of Behavioral Neurosciences & Psychology, Fairfield, USA

This article has been corrected as follows: A number of studies cited in this article (references 27 and 28) assessed a probiotic formulation previously known as VSL#3. The probiotic formulation that was assessed in these studies is now known by the generic name 'De Simone Formulation' (DSF). The current product known as VSL#3® is not the same formulation as the De Simone Formulation or the pre-2016 VSL#3. The De Simone Formulation is available under the brand names Visbiome (USA) and Vivomixx (Europe). As a result, the article has been corrected to accurately refer to this formulation as the 'De Simone Formulation'.

